# Nurses' perception of threats to human dignity in caring for patients with COVID-19: A qualitative study

**DOI:** 10.1016/j.heliyon.2024.e39983

**Published:** 2024-10-30

**Authors:** Amirheidar Bakhshiarab, Marjan Mardani Hamooleh, Akram Salamat, Seyedmohammad Mirhosseini, Ali Abbasi

**Affiliations:** a Department of Nursing, School of Nursing and Midwifery, Shahroud University of Medical Sciences, Shahroud, Iran; b Nursing and Midwifery Care Research Center, Department of Psychiatric Nursing, School of Nursing and Midwifery, Iran University of Medical Sciences, Tehran, Iran; c Department of Critical Care Nursing, Imam Khomeini Hospital Complex, Tehran University of Medical Sciences, Tehran, Iran

**Keywords:** Nurse, COVID-19, dignity, Care

## Abstract

**Purpose:**

Identifying threats to patients' dignity is crucial for maintaining and promoting their dignity. Preserving patient dignity should be regarded as both a patient’s right and a moral obligation. This study was conducted to evaluate nurses' understanding of threats to human dignity in the care of patients with COVID-19.

**Methods:**

This qualitative study was conducted using conventional content analysis in various clinical departments in Iran. Clinical nurses from different departments were selected through purposeful sampling. Data were collected via in-depth semi-structured interviews with 15 clinical nurses over two months in 2021 and analyzed using qualitative content analysis.

**Results:**

During data analysis, two main categories and ten subcategories were identified: 1) Unethical behaviors (lack of respect for patient privacy, lack of patient involvement in decision-making procedures, ignoring the patient as an individual, aggressive behaviors, stigmatization, and failure to provide palliative and end-of-life care to patients and their families); 2) Organizational factors (inadequate financial support, lack of patient-care facilities, strict restrictions, and employing less experienced nurses in COVID wards).

**Conclusion:**

The present study showed that most participants expressed concerns about violations of patients' dignity and identified many threats to it. Based on the findings, it is suggested that other factors threatening patients' dignity be identified, the threats eliminated, and healthy treatment environments created to promote patients' dignity.

## 1 Introduction

Human dignity has been important to various philosophies and religions throughout the history of mankind [1]. This concept has deep roots in ancient texts, schools, and religions, and aligns with all principles of biomedical ethics [2]. Human dignity is one of the fundamental principles and rights of all human beings [3]. Humans have a special and unique nature that underscores the high value of human dignity [4]. Human dignity is an intrinsic value [5] and is categorized into four types: merit dignity, which is based on social rank and official positions in life and exists in degrees that can fluctuate over time; moral dignity, which derives from a person's moral actions and can be diminished or destroyed through immoral actions, tied to the idea of dignified character and virtue, and unevenly distributed among people; identity dignity, which relates to the integrity of one's body and mind and often, though not always, to one's self-image, varying based on others' actions and changes in one's body and mind; and Menschenwürde, the inherent universal dignity of all human beings, equal among everyone, and which cannot be lost as long as the person exists [6]. In the medical service system, the social aspects of patients' dignity are threatened and harmed for various reasons, such as inappropriate treatment, humiliation, and organizational problems [7]. In Iran, according to the constitution, human dignity is a principle and law that is upheld at all stages of health and treatment services, ensuring respect and observance of laws for everyone [8]. Observing and respecting human dignity is an intrinsic concept and value that all medical personnel and healthcare providers should continuously strive to uphold when delivering services [9].

Respecting and preserving human dignity fosters satisfaction and happiness in patients, who should not be harmed in the process of upholding this value [10]. Failure to respect human dignity during the provision of care and healthcare services undermines patient recovery and treatment [11]. Ostaszkiewicz et al. (2020) demonstrated a direct and significant relationship between patient satisfaction with their care process and respect for their human dignity [12]. Factors that threaten the human dignity of hospitalized patients include violations of privacy [9], lack of independence [13], non-compliance with ethical and professional behavior by healthcare staff/professionals [14], lack of confidence and concern about treatment [15], insufficient support from healthcare staff/professionals [16], and breaches of confidentiality [17]. Disregarding the human dignity of patients leads to consequences such as mistrust, discomfort, and stress, all of which negatively impact patients' health [18,19]. Preservation of privacy is one of the most important aspects of human dignity, and therapeutic environments can sometimes threaten this privacy [20]. Ensuring patient privacy is essential for creating an effective and trustworthy relationship between the patient and healthcare staff/professionals; failure to do so can cause anxiety, reduce the quality of treatment, and lead to patient aggression [21]. As health status becomes unstable, especially after a life-threatening disease, patients experience mental, social, and physical injuries and disorders that jeopardize their dignity [22]. The COVID-19 pandemic is an emerging disease that has created many problems for health and treatment systems and patients. Due to the rapid spread of this disease, there are international concerns about psychiatric and psychological services for the general population [23]. The conditions resulting from the pandemic, including quarantine, isolation, social distancing, and restrictions on providing healthcare services, have challenged and threatened the moral principles and human dignity of patients [24,25]. This pandemic has impacted all aspects of nursing and the healthcare system, leading to a shortage of nurses and concerns about providing quality care services [26]. Organizational problems such as lack of nursing staff, increased patient numbers, shortage of medical equipment, increased work pressure on nurses, and reduced face-to-face interaction between nurses and patients have led to decreased quality and provision of medical services, and also threaten patients' dignity [27,28]. Due to these special conditions, patients often feel that their care is inadequate and their dignity is not respected, contributing to depression, anger, anxiety, and threats to their dignity [29]. Another significant factor threatening the dignity of these patients is related to their families, who, due to the disease's conditions, are unable to visit and provide emotional and psychological support, further compromising the patients' dignity [30].

Preserving and emphasizing human dignity is a key goal and moral principle in care [31]. Human dignity is a fundamental behavioral and moral requirement in nursing, and its importance is underscored by all nursing standards [32,33]. Valuing the rights and human dignity of patients is one of the most important values and moral obligations in the nursing profession [31]. Improving the quality of medical care and services is a top priority; therefore, valuing and preserving the human dignity of patients is crucial [34]. Despite the close relationship between the concept of human dignity and patient care, there is a lack of comprehensive definitions and sufficient knowledge about it, as well as a lack of studies on this concept. Thus, this study aims to evaluate nurses' understanding of threats to human dignity in the care of patients with COVID-19.

## 2 Materials and methods

### 2.1 Study design and participants

The current research was a qualitative study conducted using the conventional content analysis approach. Content analysis is a method of analyzing written, spoken, or visual messages about a concept. In conventional content analysis, when there is limited information about a concept, the concept under study is identified from the data text using categories and their names [35].

This study was conducted on Iranian nurses in the inpatient COVID-19 wards of medical teaching centers. A purposive sampling method was used to select participants, ensuring maximum diversity in sex, age, education level, rotating shift, general nursing work experience, and experience in caring for COVID-19 patients until data saturation was reached. Purposive sampling is used in qualitative studies to identify and select rich information for more effective use of limited resources [36].

The sample selection process was based on the following inclusion criteria: 1) full-time employment, 2) at least one month of work experience in caring for patients with COVID-19, and 3) willingness to participate in the study. A total of 15 nurses participated, meeting the inclusion criteria. The nurses were working in both general and specialized COVID-19 departments, with experience caring for COVID-19 patients ranging from 1 to 6 months. Most of the nurses had a bachelor's degree, while the rest had a master's degree. Other characteristics of the participants are provided in Table 1.

**Table 1:** Main characteristics of the participants.

Participant	Age (year)	Gender	Marital status	Working departments	degree of education	Work experience (year)	Duration of patient care with COVID-19 (month)
1	41	F	Married	ICU	BSN	18	4
2	32	F	Married	ICU	MScN	10	4
3	37	F	Married	Infectious	BScN	12	2
4	37	M	Married	ICU	MScN	12	5
5	29	F	Single	Infectious	BScN	6	4
6	37	F	Married	Emergency	BScN	11	6
7	29	F	Single	Infectious	BScN	7	6
8	34	M	Married	Infectious	BScN	11	2
9	34	F	Married	NICU	MScN	10	1
10	29	F	Single	ICU	BScN	6	4
11	42	F	Married	Urology	BScN	19	5
12	36	F	Married	ICU	BScN	13	4
13	30	M	Married	ICU	BScN	7	4
14	29	F	Married	ICU	BScN	6	2
15	47	M	Married	Oncology	BScN	22	6

F: Female; M: Male; BScN: Bachelor of Science in Nursing; MScN: Master of Science in Nursing; ICU: Intensive care unit.

### 2.2 Data collection

After receiving the letter of introduction to enter the research environment, coordination was made with the department supervisors, who introduced the qualified nurses (based on the inclusion criteria) to the researcher. The researcher then obtained the nurses' contact numbers from the supervisors. Due to the restrictions caused by the COVID-19 pandemic, data were collected through video and audio interviews on the WhatsApp social network, with informed consent obtained electronically from the nurses. Semi-structured in-depth interviews were conducted with 15 participants over two months in the second half of 2021 (October 3rd to September 28th). Purposive sampling continued until data saturation was reached. Each nurse was interviewed once, resulting in a total of 15 interviews. Prior to the interview, the researcher discussed the interview process with the nurses. The participants were not on shift at the time of the interview. In addition to interviews, field notes were used for data collection. The interview questions were based on the researchers' clinical experiences (from the perspectives of the first and third authors who worked in the COVID-19 wards) and the researchers' experiences in qualitative studies.

The focus of the interview questions was the nurses' experiences with dignity in patient care, beginning with a general question: 'How do you, as a nurse, describe dignity in caring for a COVID-19 patient?' The interviews then proceeded in a more specific manner. In this context, the nurses were asked:

• "What factors do you think can threaten the dignity in caring for the patient?"
• "What factors do you think can threaten the dignity in caring for the patient of COVID-19?"

We used such open questions as “What do you mean by this?” “Please elaborate on it”, and “Please give an example” to obtain more proper answers. In fact, the participants began to speak freely and by asking probing questions at the right time, we advanced the interview towards clarifying the phenomenon under study. (Appendix 1). The interviews lasted 32–65 min (41 min on average).

### 2.3 Data analysis

In parallel with data collection, data analysis was conducted. Each interview was recorded, transcribed, and converted into text. After reviewing the interview texts several times, semantic units were extracted. Codes were then derived from these semantic units. The codes were organized into sub-classes based on their similarities and differences. Finally, the sub-classes were combined to form classes [35].

### 2.4 Data accuracy and reliability

The accuracy and stability of the data were assessed using Guba and Lincoln’s criteria (1985), which include four criteria: believability, trustworthiness, verifiability, and transferability [37]. To determine credibility, there was continuous involvement between the subject and the data. The opinions of the research team were used throughout the data collection and analysis processes. If any team member encountered a problem during data analysis, they resolved it through consultation with the others. For example, the team's opinions were used to name categories and sub-categories, and if there was an issue with labeling, the data was reviewed again. The results were discussed with some participants, including two doctoral-level nurses and two master's-level nurses. To determine reliability, an external observer familiar with both the clinical environment and qualitative research, but not part of the research group, was used, and agreement about the results was reached. To ensure verifiability, all activities were recorded, and a report of the research process was prepared. For transferability, the results were discussed with two nurses outside the study who had experience caring for COVID-19 patients, and the results were confirmed.

The third author participated in data collection, the first and fourth authors were involved in deciphering, and the second and fifth authors were responsible for coding and creating themes. To ensure complete and transparent reporting, the information presented adheres to the COnsolidated criteria for REporting Qualitative research (CORE-Q) checklist, a widely recognized tool for standardizing research reporting in qualitative studies [38].

### 2.5 Ethics subsection

This study received approval from the Ethics Council in Biomedical Research at Iran University of Medical Sciences (IR.IUMS.REC.1399.1038). Participants were fully informed of the study’s objectives and participation conditions at the beginning of the research. The authors followed the guidelines of the Committee on Publication Ethics (COPE) in the dissemination of findings, and written informed consent was obtained from all participants.

## 3 Results

Table 2 displays the results of the data analysis, which revealed two primary categories and ten sub-categories. The primary categories include: unethical behaviors, involving lack of respect for patient privacy, lack of patient involvement in decision-making procedures, ignoring the patient as an individual, aggressive behaviors, stigmatization, and failure to provide palliative and end-of-life care to patients and their families; and organizational factors, involving inappropriate financial support, lack of patient-care facilities, strict restrictions, and employing less experienced nurses in COVID wards.

**Table 2:** Factors threatening human dignity in the care of patients with COVID-19.

Categories	Sub-categories
Unethical behaviors	Lack of respect for patient privacy
	Lack of patient involvement in decision-making procedures
	Ignoring the patient as an individual
	Aggressive behaviors
	Stigmatization
	Failure to provide the patients and their families with palliative and end-of-life care
Organizational factors	Inappropriate financial support
	Lack of patient-care facilities
	Strict restrictions
	Employing less experienced nurses in COVID wards

Participants identified unethical behavior as a potential source of disgrace for COVID-19 patients during care. They reported feeling that their own dignity was at risk if they contracted COVID-19. These behaviors include:

**A-1 Lack of respect for patient privacy:** The majority of participants believed that during the COVID-19 outbreak, patients' unique conditions— involving unconsciousness or inability to care for themselves—occasionally led to a disregard for their privacy, which poses a serious risk to their dignity during treatment. In this context, the nurses said:

"Moving COVID-19 patients on ventilators in the ICU violates their privacy and endangers their dignity because they are unconscious and their privacy is not respected." (Participant 4)

"A patient diagnosed with COVID-19 was admitted to the infectious disease department and experienced symptoms of urine incontinence. The absence of adherence to patient privacy protocols poses a significant risk to the preservation of the patient's dignity. " (Participant 5)

**A-2 Lack of patient involvement in decision-making procedures**: The lack of patient self-care led to their exclusion from decision-making processes, depriving them of any authority in determining their own treatment and care procedures. One of the study participants expressed the following viewpoint:

"When the patient is excluded from the treatment process and lacks autonomy in selecting their therapy, or when the initial rapport between the nurse and the patient is not created, the patient becomes susceptible to vulnerability and their dignity is compromised." (Participant 8)

" I think it's quite unprofessional to provide services without regard for the patient, to act without their knowledge or consent, or to skip asking them about their treatment." (Participant 2).

**A-3 Ignoring the patient as an individual:** The participants believed that they should always be treated with respect and dignity during their care. Unfortunately, this was often disregarded during the COVID-19 pandemic. In this regard, a few participants said:

"The patient's dignity is violated, for example, if a nurse approaches them directly and takes their blood without giving them any explanation or engaging in conversation." (Participant 1).

"A COVID-19 patient who is worried and anxious owing to the unknown nature of the condition has to be reassured by the nurse, yet physical restraint would be inhumane." (Participant 17).

**A-4 Aggressive behaviors**: The participants believed that the aggressive behavior of some nurses during care is a violation of their rights. One of the nurses said:

"Due to workload strain brought on by the sick leave, some nurses became irrationally angry and aggressive, yelling at patients without realizing it and endangering their dignity." (Participant 2).

**A-5 Stigmatization**: Participants stated that being stigmatized, or being accused of having a disease, was one of the most important threats to patient’s dignity during caregiving. Regarding this topic, a few respondents said:

"The patient's dignity is violated when they are blamed for their illness because of their recent actions, such as going out or attending a party." (Participant 12).

"One time, I listened in on a conversation between two of my coworkers about the patient in bed 10, who had gone to a party and was now ill and causing problems. The patient heard this and subsequently claimed he had not left the house and had no idea how he had contracted the disease." (Participant 15).

**A-6 Failure to provide palliative and end-of-life care for the patients and their families:** Participants stated that due to the high mortality rate associated with COVID-19, end-of-life care should be prioritized. However, not paying enough attention to patients is an insult to their dignity. In this regard, some of the nurses said:

"A dignified death is essential for COVID-19 patients in their final stages of life, and the patient's right to privacy must be upheld at all costs." (Participant 2).

"There are several potential dangers to patients' dignity in the special care unit; for instance, when an elderly patient is close to death, no serious attempts are made to revive him." (Participant 12).

### 3.1 B- organizational factors

Organizational issues were a threat to patients' dignity during the COVID-19 pandemic. These included inappropriate financial support, lack of patient-care facilities, strict restrictions, and the employment of less experienced nurses in COVID wards. These factors are as follows:

**B-1 Inappropriate** financial support**:** Participants believed that one of the main factors undermining patients' dignity was inappropriate organizational financial support, especially for the COVID-19 treatment. One of the nurses said:

"Most elderly patients are retired, and without insurance, they have to shoulder heavy financial burdens that can put them in a state of anxiety and even compromise their dignity." (Participant 12).

**B-2 Lack of patient-care facilities**: The participants stated that insufficiency of supplies, equipment and essential medications for COVID-19 within the hospital setting has resulted in numerous patient challenges, which poses a significant risk to providing quality treatment and will endanger human dignity. Regarding this matter, certain individuals expressed:

"The presence of organizational challenges, such as insufficient bed availability, oxygen supply issues, and pharmaceutical shortages, can elicit feelings of anxiety and discomfort in patients. Consequently, patients may begin to question the hospital's legitimacy due to its inability to provide necessary medications. Patients' dignity is at risk due to these factors." (Participant 9).

"COVID-19 patients are typically hospitalized for a week with no visits, no special entertainment, and no television in the room, all of which threatens patients' dignity due to boredom and perplexity." (Participant 16).

**B-3 Strict restrictions**: Participants believed that organizational strict regulations can hinder nurses' ability to provide quality care to their patients. In this regard, some nurses said:

"A lot of work pressure and financial troubles for nurses causes them to get demotivated and agitated, which immediately causes inappropriate interaction between the nurse and the patient, as well as not performing care duties effectively and precisely, compromising the patient's dignity." (Participant 7).

"Visiting restrictions for COVID-19 patients in the intensive care unit can extend for up to a month, adding stress to an already difficult situation and putting the patient's dignity at risk." (Participant 11).

**B-4 Employing less experienced nurses in COVID wards**: The participants stated that employment of low experience nurses in the COVID wards causes many problems. Therefore, the patients are dissatisfied, and their dignity is damaged. According to several respondents:

"Patients' privacy and reputation are at risk when medical staff, do not have enough experienceto deal with a new condition, fails to provide them with proper information." (Participant 12).

"When patients with COVID-19 were first admitted during the outbreak, nurses sometimes avoided talking to them out of concern of spreading the disease. In addition, nurses' lack of knowledge of the illness meant that they could not address patients' concerns, which in turn put patients' dignity at risk." (Participant 4).

## 4 Discussion

The participants' accounts of incidents in which COVID-19 patients' human dignity was threatened led them to classify these threats into two broad categories: unethical behaviors and organizational factors. This study found that contracting COVID-19 undermines patients' dignity when receiving care. Upholding human dignity involves freedom, responsibility, duty, and service to others, and is based on protecting human rights. All human beings have inherent worth and dignity, regardless of their condition or position. According to Silva et al., 'dignity' is both a human value and an expression; as an internal and intrinsic value, it is the foundation of each person's uniqueness and the reason for respecting them. Good behavior and values like kindness, respect, conscience, and ethics are also essential and foundational to human dignity [39]. A previous study showed that the COVID-19 pandemic poses a significant threat to human dignity, particularly for patients in various healthcare settings, including palliative care, medical and surgical units, intensive care, and long-term care facilities [40].

Within the category of 'unethical behaviors,' several sub-categories were identified: lack of respect for patient privacy, lack of patient involvement in decision-making procedures, ignoring the patient as an individual, aggressive behaviors, stigmatization, and failure to provide patients and their families with palliative and end-of-life care.

Disregarding patient privacy emerged as a significant threat to patient dignity. In this context, Fuseini et al. reported that patient and nurse experiences suggest that a disregard for patient privacy has a direct negative impact on patients' human dignity [20]. According to Clancy et al., the degree to which patients' privacy is protected closely correlates with the quality of treatment they receive [18]. Based on a study by Martinez et al., respecting patients' privacy not only reduces their distress and anxiety but also fosters positive connections with healthcare providers. This, in turn, allows for the open discussion of crucial treatment matters that are important for both the patient and the healthcare team [41]. Beckstrand et al. revealed a significant association between the lack of privacy for patients and inadequate medical treatment outcomes [42]. In this context, the results of the current study align with the existing body of literature.

Lack of patients' participation in decision-making processes threatened their human dignity. Communication and engagement in their treatment affect patients' human dignity [31]. In this regard, Aydn et al. show that a lack of understanding regarding medical diagnoses endangers patients' dignity [43]. Similar findings were also observed in two Pakistani public and private hospitals (by Humayun et al. and Mukhtar et al., respectively) [44,45], with 90 % of public hospital patients and 53.3 % of private hospital patients reporting they did not have the right to participate in their treatment. Factors such as patients' age, culture, and language can impede communication and participation in their therapy. Agyemang-Duah et al. [46] found that non-participation in decision-making is primarily due to linguistic barriers, advanced age, and illiteracy.

Disregarding the patient's humanity was shown to be a significant threat to human dignity in this investigation. Nurses have the most contact with patients and, therefore, the greatest impact on treatment [40]. The way nurses treat patients can greatly influence whether the patient feels respected or threatened by the staff. Cusack et al. [47] found that nurses often focus on their equipment rather than their patients during conversations, fail to make eye contact, and use degrading terms when addressing them. Abraham et al. found that patients' dignity is severely compromised due to nurses' poor communication skills [48]. The findings of the present study are consistent with mentioned studies.

Another major threat to patients' dignity in this study was aggressive behavior. Patients' dignity is threatened by various unethical behaviors, including neglect and humiliation [49,50], verbal threats, verbal and nonverbal harassment, and insults based on race or religion by nurses or doctors [27]. Furthermore, disdain towards patients and their families compromises their dignity and lowers their self-worth [50]. Antoniazzi found that several harmful behaviors, particularly in professional interactions between nurses and patients, are characterized by a lack of respect for personal dignity. These behaviors include violence, anger, verbal abuse, and aggression toward patients [51]. The presented previous studies are consistent with the findings of the current study.

The findings of this investigation also indicate that stigmatization is a significant factor threatening the dignity of patients. Arshak et al. [52] showed that 85 % of COVID-19 patients experienced stigmatization. Imran et al. [53] demonstrated that individuals diagnosed with COVID-19 encountered significant social stigmatization. This stigmatization often manifests through symptoms such as cough, fever, and other commonly associated signs of the disease. It is sometimes accompanied by discrimination and derogatory comments, including accusations of irresponsibility, failure to adhere to hygienic practices, thoughtlessness, and carelessness [54]. Ramaci et al. showed that stigmatization and negative attitudes towards COVID-19 patients cause them to feel judged and diminish their dignity [55]. In this regard, the findings of the present study are consistent with the existing literature.

The current study's findings indicate that the lack of palliative care and end-of-life support for patients and their families poses a significant risk to patients' human dignity. Patient care takes on particular significance in the latter stages of life, encompassing a broad range of services such as pain management, family support, and the administration of life-extending medications [56]. According to Bovero et al.'s study, patients in this stage require deferential and meaningful behavior, which is essential for upholding and respecting human dignity [57]. The preservation of patients' dignity at the end of life is directly correlated with maintaining their privacy and respect [58]. Xiao et al.'s study shows that respecting patients' privacy, treating them with decency, and honoring their family's wishes all contribute to the patient's sense of worth [59]. Health and treatment personnel must be aware of the phases and situations in which patients' lives are ending and assist the patients' families in maintaining their dignity to promote and enhance the patients' dignity [60]. These findings are consistent with the previous studies.

Sub-categories of "organizational factors" included inappropriate financial support, lack of patient-care facilities, strict restrictions and employing less experienced nurses in COVID wards.

The findings of this study suggest that inadequate financial support is a major factor undermining patients' human dignity. Patients should receive care regardless of their socioeconomic or cultural background [39]. Chen et al. [61] found that patients' dignity is protected and their communication improved when healthcare workers avoid making assumptions about their ability to pay for treatment. According to research conducted by Alikari et al., healthcare providers are less likely to respect the human dignity of financially disadvantaged patients [62].

Another significant challenge to human dignity identified in this study was the inadequate provision of medical facilities for patients. Some organizational elements can compromise the dignity of patients diagnosed with COVID-19 during their medical treatment. The lack of essential resources, such as oxygen, blankets, and bed linens, hinders patients from effectively utilizing the available healthcare amenities. The shortage of sufficient beds and institutional equipment results in patients being admitted to wards characterized by high levels of noise and poor cleanliness, which compromises their human dignity. A study conducted in Spain found that nurses expressed concerns about the insufficient space and inadequate equipment in hospital wards, which they said threatened the preservation of patients' human dignity [63]. Yalden and McCormack [64] discovered that healthcare and treatment facilities significantly impact the promotion of professional dignity among nurses. It should be noted that Iran's current economic struggles, compounded by shortages of personal protective equipment, healthcare workers, and facilities, have made it difficult to uphold the dignity of COVID-19 patients [40]. In this respect, the findings of the present study are in agreement with the literature.

Strict organizational norms were another threat to patients' dignity. An appropriate workload is one of the organizational qualities that support human dignity [65,66], leading to reduced nurse burnout and a more satisfying work environment. Conversely, lack of time and a heavy workload [49,65,67,68] are among the most serious challenges to dignity, as they divert nurses' attention from their primary role of patient care and endanger patients' dignity [67]. Due to increased workloads, many nurses have reported a lack of respect for patient dignity. Consequently, many have been forced to transfer departments or even leave their careers due to workplace violations of dignity [49]. The COVID-19 condition also prevented patients from meeting their companions, which further harmed their dignity. In this context, Son et al. found that inadequate support in the treatment environment, along with strict regulations, threatened patients' dignity [69]. The results of the current study are in line with those of previous research.

Another challenge to patients' human dignity was the employment of less experienced nurses in COVID wards. Numerous studies have highlighted the importance of nurses' professional skills and expertise. Nasrin et al. [70] asserted that inadequate qualifications lead to several challenges for both nurses and patients, ultimately resulting in the erosion of dignity. Valizadeh et al. found that nurses should strive to project a professional image that reflects their esteemed standing. This can be achieved by enhancing their qualifications and scientific expertise, which helps address any misconceptions about their profession in both the media and public perception. Insufficient knowledge and skills pose a significant risk to patient dignity [49]. The use of novice nurses in specialized departments early in their careers has contributed to their exhaustion and dissatisfaction, potentially compromising patient care and risking their sense of dignity. Khademi et al. reported that novice nurses face numerous challenges due to inadequate knowledge, which threatens patient dignity and can lead to lasting consequences [50]. The findings of this study align with the results of earlier research.

To fully understand the study's conclusions, it is crucial to consider its inherent limitations. The data collection was confined to a specific geographic area within Iran, focusing on a single city. Therefore, there may be different threats to patient dignity in other locations and outside of Iran. Another limitation of this study was the reliance on the subjective perspectives of participants in a qualitative investigation to define the notion of patient dignity. As a result, employing alternative research methodologies could provide further insights into this concept. Additionally, further exploration is needed to examine threats based on the experiences of clinical nurses.

## 5 Conclusion

The results of the current study indicate that there are two distinct categories of factors threatening patients' dignity: unethical behaviors and organizational factors. Patients' dignity is at risk in various settings, including the work environment, organizations, society, and the family unit. Therefore, identifying these threats and working to mitigate them is crucial for creating a healthier treatment environment and improving patients' lives. Identifying factors that compromise patient dignity is essential for guiding nursing managers and organizations. This understanding can inform the development of interventions to address these threats and foster a work environment that upholds patient dignity.

Additionally, future studies should explore threats to dignity in the care of both acute and chronic conditions. Research should also include non-clinical nurses working in diverse settings, as well as other healthcare professionals such as physicians and physiotherapists.

## CRediT authorship contribution statement

**Amirheidar Bakhshiarab:** Writing – review & editing, Writing – original draft, Project administration, Investigation, Conceptualization. **Marjan Mardani Hamooleh:** Writing – review & editing, Writing – original draft, Formal analysis, Conceptualization. **Akram Salamat:** Writing – review & editing, Writing – original draft, Investigation. **Seyedmohammad Mirhosseini:** Writing – review & editing, Writing – original draft, Formal analysis. **Ali Abbasi:** Writing – review & editing, Writing – original draft, Supervision, Conceptualization.

## Data availability

Information will be available upon request from the corresponding author.

## Funding

This research did not receive any specific grant from funding agencies in the public, commercial, or not-for-profit sectors.

## Declaration of competing interest

The authors declare that they have no known competing financial interests or personal relationships that could have appeared to influence the work reported in this paper.
