# Supplementary material for: Nurses' perception of threats to human dignity in caring for patients with COVID-19: A qualitative study
Source: Heliyon. 2024 Oct 30;10(21):e39983. doi: 10.1016/j.heliyon.2024.e39983 (PMC11584603; doi:10.1016/j.heliyon.2024.e39983)
Supplement: Multimedia component 1 [file mmc1.docx]

**Appendix 1**

**Interview guide**

The focus of the interview questions was the nurses' experience in the field of dignity in caring, which was first a general question based on "How do you, as a nurse, describe the dignity in caring for the patient of COVID-19?" It was asked and then the interviews were conducted in a more specific manner. In this regard, the nurses were asked:

- "What factors do you think can threaten the dignity in caring for the patient?"
- "What factors do you think can threaten the dignity in caring for the patient of COVID-19?"

We used such open questions as “What do you mean by this?” “Please elaborate on it”, and “Please give an example” to obtain more proper answers. In fact, the participants began to speak freely and by asking probing questions at the right time, we advanced the interview towards clarifying the phenomenon under study.
